# Can Hemorrhagic Stroke Genetics Help Forensic Diagnosis in Pediatric Age (<5 Years Old)?

**DOI:** 10.3390/genes15050618

**Published:** 2024-05-13

**Authors:** Biancamaria Treves, Elena Sonnini, Raffaele La Russa, Fabio Del Duca, Alessandro Ghamlouch, Alessandra De Matteis, Claudia Trignano, Juan Antonio Marchal, Esmeralda Carrillo, Gabriele Napoletano, Aniello Maiese

**Affiliations:** 1 Department of Anatomical, Histological, Forensic and Orthopaedic Sciences, Sapienza University of Rome, 00161 Rome, Italy; biancamaria.treves@uniroma1.it (B.T.); fabio.delduca@uniroma1.it (F.D.D.); alessandro.ghamlouch@uniroma1.it (A.G.); alessandra.dematteis@uniroma1.it (A.D.M.); gabriele.napoletano@uniroma1.it (G.N.); 2 Dipartimento Scienze della Vita e Sanità Pubblica, Medicina Genomica, Università Cattolica del Sacro Cuore, 00168 Rome, Italy; elena.sonnini01@icatt.it; 3 Department of Clinical Medicine, Public Health, Life Sciences, and Environmental Sciences, University of L’Aquila, 67100 L’Aquila, Italy; 4 Department of Biomedical Sciences, University of Sassari, Viale San Pietro 43c, 07100 Sassari, Italy; ctrignano@uniss.it; 5 Centre for Biomedical Research (CIBM), Biopathology and Regenerative Medicine Institute (IBIMER), University of Granada, 18016 Granada, Spain; jamarchal@ugr.es (J.A.M.); esmeral@ugr.es (E.C.); 6 Instituto de Investigación Biosanitaria ibs. GRANADA, University of Granada, 18071 Granada, Spain; 7 Excellence Research Unit “Modeling Nature” (MNat), University of Granada, 18016 Granada, Spain; 8 Department of Human Anatomy and Embryology, Faculty of Medicine, University of Granada, 18016 Granada, Spain; 9 Department of Surgical Pathology, Medical, Molecular and Critical Area, Institute of Legal Medicine, University of Pisa, 56126 Pisa, Italy

**Keywords:** stroke, intracranial hemorrhage, genetic, monogenic, non-accidental head injury (NAHI), abusive head trauma (AHT), non-accidental trauma (NAT), child, forensic science

## Abstract

When stroke occurs in pediatric age, it might be mistakenly interpreted as non-accidental head injury (NAHI). In these situations, a multidisciplinary approach is fundamental, including a thorough personal and familial history, along with accurate physical examination and additional investigations. Especially when the clinical picture is uncertain, it is important to remember that certain genetic conditions can cause bleeding inside the brain, which may resemble NAHI. Pediatric strokes occurring around the time of birth can also be an initial sign of undiagnosed genetic disorders. Hence, it is crucial to conduct a thorough evaluation, including genetic testing, when there is a suspicion of NAHI but the symptoms are unclear. In these cases, a characteristic set of symptoms is often observed. This study aims to summarize some of the genetic causes of hemorrhagic stroke in the pediatric population, thus mimicking non-accidental head injury, considering elements that can be useful in characterizing pathologies. A systematic review of genetic disorders that may cause ICH in children was carried out according to the Preferred Reporting Item for Systematic Review (PRISMA) standards. We selected 10 articles regarding the main genetic diseases in stroke; we additionally selected 11 papers concerning patients with pediatric stroke and genetic diseases, or studies outlining the characteristics of stroke in these patients. The disorders we identified were Moyamoya disease (MMD), *COL4A1*, *COL4A2* pathogenic variant, Ehlers–Danlos syndrome (E-D), neurofibromatosis type 1 (Nf1), sickle cell disease (SCD), cerebral cavernous malformations (CCM), hereditary hemorrhagic telangiectasia (HHT) and Marfan syndrome. In conclusion, this paper provides a comprehensive overview of the genetic disorders that could be tested in children when there is a suspicion of NAHI but an unclear picture.

## 1. Introduction

Pediatric stroke is a cerebrovascular condition that can be subdivided based on the age of onset into perinatal stroke, which is a stroke occurring between the 28th week of gestation and the 28th day of life, and childhood stroke after the 28th day [1]. Stroke can also be classified based on the underlying cause into ischemic stroke and hemorrhagic stroke (HS). The latter includes intracerebral hemorrhage (ICH), subarachnoid hemorrhage (SAH) and intraventricular hemorrhage (IVH) [2]. In the general population, it is estimated that approximately 7% of strokes are caused by SAH, while 10% are attributed to ICH [3]. Hemorrhagic stroke and ischemic stroke in children and young adults have an incidence exceeding 1 per 100,000 children [4]. According to Armstrong-Wells et al., the prevalence of perinatal hemorrhagic stroke is estimated to be approximately 6.2 per 100,000 births [5]. Conversely, in the pediatric population, hemorrhagic stroke has an incidence ranging from 1 to 1.7 per 100,000 per year, representing approximately half of pediatric strokes [1]. The incidence rate of non-traumatic subarachnoid hemorrhage (SAH) is estimated around 0.4 per 100,000 per year and the rates of intracerebral hemorrhage (ICH) in pediatric patients is 0.8 per 100,000 per year [6]. Intracranial hemorrhage may also be related to the presence of arteriovenous malformations (AVM), which is the most common cause of pediatric ICH. Stroke represents one of the ten leading causes of infant mortality [7]. However, only a small percentage of strokes can be attributed to genetic diseases, estimated at around 1%; this percentage is higher if we only take into account pediatric strokes [8]. It is important to remember that when stroke occurs due to a monogenic disease, it could be accompanied by a unique set of symptoms. Some genetic diseases that result in intracranial hemorrhages (intraparenchymal, subdural hemorrhages (SDH), subarachnoid, optic chiasm/retinal hemorrhages) in early age might be mistakenly interpreted as non-accidental head injury (NAHI). Therefore, when there is a suspicion of an abusive head trauma (AHT) or non-accidental head injury (NAHI), it is important to gather current and family history, as well as medical information pertaining to the child (including objective, radiological and laboratory examinations). A multidisciplinary diagnostic approach that considers morphological and syndromic characteristics—in addition to clinical manifestations—can be useful for selecting the most appropriate genetic test and for understanding whether the injuries sustained by the child are wholly or partially attributed to a genetic disease rather than NAHI [9]. NAHI and AHT are significant issues worldwide that often involve children under 5 years of age. Classically, the clinical manifestations of NAHI include subdural hematoma (SDH), encephalopathy and bilateral retinal hemorrhage—also considered the classic triad—which can be associated with fractures and other bodily injuries. However, there may be cases where homicide is not intentional but is caused by the exaggerated actions of an adult who cannot handle a child’s behaviors (e.g., crying).

Sometimes the suspicion of NAHI arises in the presence of fractures or minor external injuries from falls, or in cases of intracranial bleeding of dubious interpretation. In these instances, circumstantial information and clinical data are crucial to distinguish NAHI from accidental head trauma. The clinical manifestations that could potentially arouse suspicion of abusive trauma are briefly outlined in Table 1 [10,11]. In cases of unexplained fractures and intracranial hemorrhages in young children, genetic diseases that could cause clinically ambiguous manifestations should be ruled out. It is important to highlight the dysmorphic features that emerge upon inspection of the face and body. Therefore, a multidisciplinary evaluation (forensic physician, pediatrician and geneticist) can be extremely useful in framing the case. In this review, we have examined genetic diseases that can cause hemorrhagic stroke in children and that without careful investigation could be misinterpreted as NAHI. Many etiologies of HS are well understood; some are only potential associations. Often HS remains idiopathic, although many children have identifiable risk factors, often multiple.

However, due to the vast number of genetic disorders and their ongoing discovery, it is important to continue documenting and sharing such cases while keeping in mind the limitations of this type of research.

## 2. Materials and Methods

**Figure 1:**
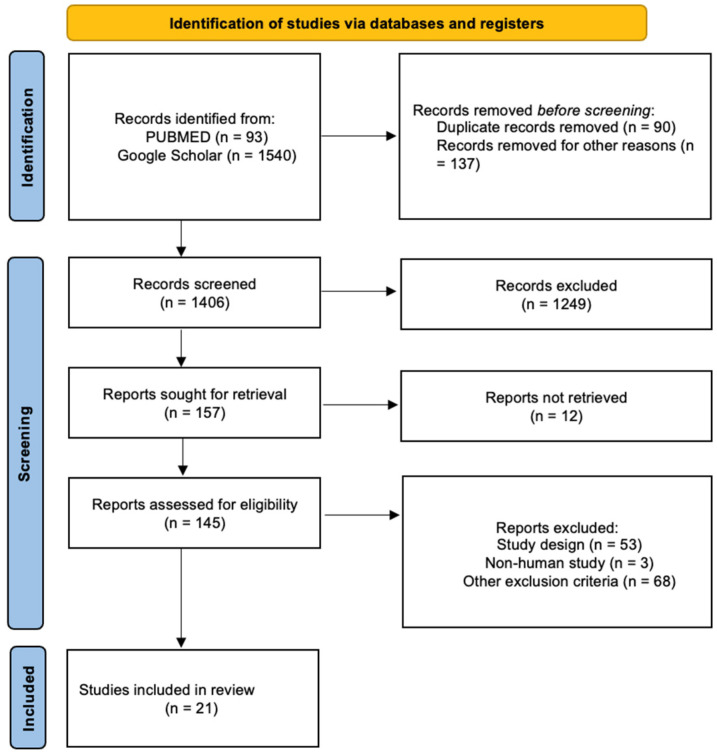
The selection of papers following the PRISMA protocol.

The present systematic review was carried out according to the Preferred Reporting Item for Systematic Review (PRISMA) standards. A methodological appraisal of each study was conducted according to the PRISMA standards, including an evaluation of bias. The PRISMA 2020 Statement was applied. It consists of a checklist and a flow diagram (Figure 1).

We performed a review of the English literature regarding genetic causes that can cause intracranial hemorrhage in children, thus mimicking non-accidental head injury.

A systematic literature search and critical review of the collected studies were conducted. An electronic search of PubMed (93) and Google Scholar (1540) from database inception to March 2024 was performed. Databases were investigated using the following research terms (“pediatric” OR “child”) AND (“autopsy” OR “death”) AND (“Haemorrhagic Stroke” OR “Intracranial Haemorrhage”) AND (“genetic” OR “monogenic”) in all fields (e.g., title, abstract and keywords).

A systematic search of the literature was conducted by reviewing the titles and abstracts, along with a manual examination of the references. We reviewed the reference lists of all the articles we identified to ensure that we did not miss any relevant literature.

From this research, a list of abstracts was organized in the form of a dataset and all the dataset was downloaded in a .nbib file and uploaded to Software Zotero 6-0.30, used as a citation manager. 

The research group, following a meeting, established the inclusion and exclusion criteria for papers, in accordance with the PRISMA standards.

First of all, two investigators (B.T and G.N.) read all the abstracts found from the databases. The bibliographies of all identified papers were examined and cross-referenced to further identify relevant literature. After selecting abstracts and investigating the bibliographies of related papers, data collection began. One investigator (B.T.) independently examined papers with titles or abstracts that appeared to be relevant, selecting those that analyzed the HS or intracranial hemorrhage due to genetic disease in children. Adult or young adult subjects were excluded, to restrict our search to pediatric age (<5 years old).

The data collection process included study selection and data extraction. Disagreements concerning eligibility among the researchers were resolved by consensus. Only papers in English were included.

Data extraction was performed by two investigators (B.T. and G.N.) and verified by additional investigators (A.M. and R.L.R.).

The current study provides a useful overview for those studying genetic conditions that are associated with hemorrhagic stroke.

### Quality Assessment

The evaluation of quality (risk of bias) was conducted by two independent reviewers (A.G. and B.T.), using various scales depending on the study design. Disagreements were solved through comparison between the two reviewers.

First, for case reports and case series we utilized the assessment tool developed by Murad et al. for appraising the quality of case reports/case series, which comprehends four domains: selection, ascertainment, causality and reporting [12]. To summarize the results of each paper, Murad et al. suggest not to use an aggregate score, but instead to make a comprehensive assessment. Four studies were evaluated as “fair”, and two studies were evaluated as “good”.

For the assessment of the risk of bias in retrospective observational studies, we employed the Newcastle–Ottawa Scale (NOS) for cohort study, which includes three domains: selection (maximum 4 points), comparability (maximum 2 points) and outcome (maximum 3 points) [13]. The final sum of scores can reach a maximum of nine points. The scores can be categorized as follows: low (≤4 points), moderate (five or six points) or high (≥7 points). In our scenario, the utilization of this scale was constrained by the tool’s original design for cohort study evaluation, while our studies were descriptive studies without a control group. Not applicable (N/A) answers were assigned 0 points. As a consequence, the final scores have been inevitably impacted. Nevertheless, the NOS scale appeared the most appropriate and it is widely used in the scientific literature. Three studies scored ≤ 4 points, while two studies scored 5 or 6 points.

The quality assessment for the case-control study was performed using the Newcastle–Ottawa Scale (NOS) for case-control study [13]. It explores three areas: selection (maximum 4 points), comparability (maximum 2 points) and outcome (maximum 3 points) [13]. The final score can be classified as the latter scale. The included case–control study scored a total of six points, equivalent to a moderate risk of bias.

Finally, despite the lack of formal instruments to evaluate risk of bias in narrative reviews, we utilized the Scale for the Assessment of Narrative Review Articles (SANRA) [14], which we also utilized for evaluating the scientific statement by the American Heart Association [1]. The scale is formed by six items, each rated from 0 to 2, the high standard being 2 while 0 is a low standard score. Therefore, the maximal sum score is 12. We interpreted the total score as follows: low (≤3 points), moderate (4 to 8 points) or high (≥9 points). Only one paper scored three points, whereas all the other studies’ scores fell within the “moderate” category.

Quality assessment of the included studies is shown in Supplementary Table S1.

## 3. Results

This systematic search of the literature identified 157 papers, which were then screened based on their abstract. Among the resulting 157 articles, 12 were not retrieved, which left 145 articles for further consideration.

Papers in a non-English language were excluded (total articles 137), and the following inclusion criteria were used: (1) original research articles, (2) reviews and mini-reviews and (3) case reports/series. The mentioned publications were meticulously assessed, taking into account the primary objectives of the review. This evaluation left 21 scientific papers comprising original research articles, case reports and case series.

The included articles have been divided into two different groups, which are outlined in Table 2 and Table 3.

### 3.1. Genetic Diseases in Stroke

In Table 2, we provide a summary of the main articles (10 articles) that address genetic diseases in stroke. After reviewing the genes mentioned in the studies, we subsequently listed those that may be tested in a child who has experienced hemorrhagic stroke. Other genes that were mentioned in the articles were not taken into account, as they are more commonly associated with ischemic stroke or stroke in adult patients, which did not align with the aim of this work.

Regarding genetic disorders that may cause strokes, COL4A1 mutation was mentioned in nine articles, while COL4A2 was mentioned in four articles. Four studies cited hereditary hemorrhagic telangiectasia (HHT) or the associated mutations, and four studies referred to genetic cerebral cavernous malformations (CCM), or to its mutated genes. In addition, Marfan syndrome, Moyamoya and neurofibromatosis type 1 (NF1) were mentioned in four papers and Ehlers–Danlos syndrome (E-D) in five articles. Among these studies, only one cited sickle cell disease (SCD).

### 3.2. Stroke in Children and Genetic Disorders

Table 3 contains articles reporting case reports or case series of patients with a history of pediatric stroke in whom monogenic disease was investigated and/or diagnosed, or studies outlining the characteristics of stroke in these patients.

Two studies concern children with CCM, aged 18 months and 3 years old, presenting with bleeding cavernomas; in addition, in the first one several silent cavernomas were also detected [23,24].

There are two articles regarding patients with E-D syndrome. In the first one, E-D type IV was investigated in a child in whom trauma was suspected as the subdural hemorrhage was associated with retinal hemorrhages and bone fractures [9]. The second article regards the E-D Kyphoscoliotic-type; the patient was 13 years old at the time of the study yet reported a history of hemorrhage since birth [25].

The reported paper about Marfan syndrome is about neurovascular complications and cerebral small vessel disease in affected patients [26].

In addition, three studies concerning the risks of cerebrovascular pathology in neurofibromatosis type 1 in children are reported [27,28,29].

Finally, a study reported three children with SCD, aged 2.5, 4 and 6 years old, who suffered non-traumatic intra-parenchymal hemorrhage [30].

**Table 3:** Pediatric stroke and genetic.

Title	Authors	Year	Genetic Disease	Age	Sex
Stroke in Ehlers–Danlos Syndrome Kyphoscoliotic Type: Dissection or Vasculitis?	Quade et al. [25]	2017	E-D	Stroke episode at 13 yo, but history of bleeding since birth	F
High mutation detection rates in cerebral cavernous malformation upon stringent inclusion criteria: one-third of probands are minors	Spiegler et al. [23]	2014	CCM	18 months	M
Increased Risk of Cerebrovascular Disease Among Patients With Neurofibromatosis Type 1: Population-Based Approach	Terry et al. [27]	2016	Nf1	Children	N/A
Intracranial hemorrhage in infants and children with hereditary hemorrhagic telangiectasia (Osler-Weber-Rendu syndrome)	Morgan et al. [31]	2002	HHT	Child and neonatal	F, M
Brain arteriovenous malformations in patients with hereditary hemorrhagic telangiectasia: clinical presentation and anatomical distribution	Saleh et al. [32]	2013	HHT	Children, 3 patients < 2 yo	F, M
Cerebrovascular abnormalities in a population of children with neurofibromatosis type 1	Rosser et al. [28]	2005	Nf1	Some patients < 2 yo	F, M
Neurovascular complications of Marfan syndrome: a retrospective, hospital-based study	Wityk et al. [26]	2002	Marfan	1 patient < 1 yo	F
Genetic causes of fractures and subdural hematomas: factversus fiction	Shur et al. [9]	2021	E-D, Menkes, glutaric acidemia type 1	<1 a	F
Cerebral arteriopathy in children with neurofibromatosis type 1	Rea et al. [29]	2009	Nf1	Children	F, M
Clinical impact of CCM mutation detection in familial cavernous angioma	Sürücü et al. [24]	2006	CCM1	3 yo	F
Nontraumatic Brain Hemorrhage in Children: Etiology and Presentation	Al-Jarallah et al. [30]	2000	SCD	2.5, 4 and 6 yo	N/A

**Abbreviations**: CCM: cerebral cavernous malformations, E-D: Ehlers–Danlos syndrome, F: female, N/A: not applicable, Nf1: neurofibromatosis type 1, HHT: hereditary hemorrhagic telangiectasia, M: male, SCD: sickle cell disease, yo: years old.

## 4. Discussion

From the studies selected in our systematic literature review on the subject, we have identified genetic conditions that could potentially correlate with intracranial hemorrhage (ICH) in children. The most relevant diseases that might be suspected in differential diagnosis with non-accidental head injury (NAHI) will be further described. They were Moyamoya disease (MMD), COL4A1, COL4A2 mutations, Ehlers–Danlos syndrome (E-D), neurofibromatosis type 1 (Nf1), sickle cell disease (SCD), cerebral cavernous malformations (CCM), hereditary hemorrhagic telangiectasia (HHT) and Marfan syndrome.

The evaluation of suspected NAHI cases may be complicated by the variety of genetic conditions that can lead to hemorrhagic stroke. Several bleeding disorders could potentially be confused with abusive injuries (e.g., hemophilia, von Willebrand disease (VWD), fibrinogen disorders, vitamin K deficiency, factor XIII deficiency and other factor deficiencies, thrombocytopenia, aplastic anemia and other infiltrative or bone marrow failure syndromes and abnormalities of platelet function, etc.) [33]. Most of these conditions may present with mucosal bleeding, such as epistaxis and skin bruising, but it has been noted that some (particularly factor deficiencies) present with isolated ICH or may increase susceptibility to severe ICH after minor trauma. Collagen disorders can also predispose to easy bruising/bleeding in certain circumstances. In children presenting with ICH but without other strongly indicative findings of abuse, such as significant abdominal trauma, burns, bruises or fractures, an evaluation for other medical conditions causing or contributing to the outcomes is necessary. Additionally, physicians must recognize that, although evidence of old inflicted injuries, such as healing fractures, may support the diagnosis of abuse, healing injuries may not be correlated with recent bruises or ICH. Furthermore, when non-accidental trauma (NAT) is suspected, possible genetic and metabolic causes should be ruled out [34], for example, osteogenesis imperfecta (OI) and glutaric aciduria type 1 (GA1). Diagnostic challenges can involve every aspect of the investigative phase, from clinical examination to radiological tests to post-mortem investigations [35]. Below are the characteristic aspects of some of the hereditary conditions listed in Table 3 that can lead to hemorrhagic stroke (HS)s and should be considered before suspecting non-accidental head injury (NAHI). It is important to be aware that such genetic conditions may also be present in other family members, but due to incomplete penetrance or variable expressivity, they may have never been diagnosed. This study aims to summarize some of the genetic causes of hemorrhagic stroke, considering elements that can be useful in deciphering the cause within that framework, including age of onset, ethnicity and socioeconomic status.

### 4.1. Coagulopathies

Coagulopathies encompass a spectrum of hemorrhagic disorders characterized by dysfunctions in the coagulation cascade, predisposing individuals to prolonged or excessive bleeding. These conditions may present symptoms that, if not adequately interpreted, could be erroneously attributed to signs of abusive injuries, especially in contexts involving pediatric patients. Among the most prominent coagulopathies is hemophilia, a predominantly male genetic disorder characterized by deficiency or absence of coagulation factors [36]. The susceptibility to spontaneous or trauma-related bleeding in individuals with hemophilia should be cautiously evaluated as it may be misconstrued as evidence of physical abuse. Identifying platelet dysfunction early avoids subjecting the patient to further invasive tests, thereby containing the public costs [37]. Von Willebrand disease (VWD) represents another common coagulopathy, associated with defects in the von Willebrand protein, a multimeric glycoprotein which mediates platelet adhesion and serves as a carrier of coagulation Factor VIII. Symptoms such as easy bruising, mild gastrointestinal bleeding and recurrent epistaxis in individuals with VWD may be misinterpreted as indicative of inflicted injuries. The severity of bleeding tendency depends on whether there is a complete absence or a reduction in the von Willebrand protein. Therefore, evidence of VWD could explain intracranial hemorrhages and retinal hemorrhages following minor traumas [38]. Vitamin K deficiency represents another condition that can compromise blood coagulation. Vitamin K is essential for the synthesis of coagulation factors in the liver, and its deficiency can lead to excessive bleeding, as seen in neonatal hemorrhagic disease [39]. Failure to identify and correct this condition may lead to misunderstandings regarding the origin of bleeding. Other deficiencies in coagulation factors, such as Factor XIII deficiency, can lead to hemorrhagic disorders easily mistaken for abusive injuries. Factor XIII, also known as “fibrin-stabilizing factor”, is an enzyme that creates strong chemical bonds between soluble fibrin units formed during blood clotting [40]. These dysfunctions can result in persistent or excessive bleeding, erroneously interpreted as the result of intentional trauma. A presumptive diagnosis of Factor XIII deficiency was made using a clot solubility screening test, and confirmation was achieved by demonstrating the absence of cross-linked fibrin chains via electrophoresis. Therefore, platelet reduction count or dysfunction should be considered, impairing blood clot formation [41]. In this case, it is crucial for medical professionals to consider the possibility of coagulopathies and other medical conditions influencing blood coagulation when suspecting abuse in minors. In fact, laboratory screening studies should include a complete blood count and prolonged coagulation studies, such as prothrombin time (PT) and partial thromboplastin time (PTT). Additionally, platelet function evaluation tests (e.g., prolongation of collagen/epinephrine (CEPI), collagen/ADP (CADP)) and a pediatric hematological consultation should be considered [37]. A thorough and accurate evaluation is essential to ensure early diagnosis and treatment, thereby reducing the risk of misinterpretations and serious medical consequences.

### 4.2. Moyamoya

Moyamoya disease (MMD) is a rare progressive condition characterized by the narrowing or occlusion of the distal portion of the internal carotid artery (ICA) and/or, less frequently, the stenosis of the proximal portion of the anterior or middle cerebral artery (ACA, MCA) [42]. This pattern of blood vessel occlusion and the resulting tissue hypoxia, lead to a compensatory mechanism, consisting of the formation of collateral vessels around the stenotic arteries, called “base collateral vessels” (BCs). When scanned in conventional angiography, this network typically presents as a “puff of smoke image” (which is called “moyamoya” in Japanese) [43]. This typical pattern of brain circulation increases cerebral ischemic stroke, because of reduced brain flow, and hemorrhagic stroke risk, due to the augmented fragility of BCs. It is a rare condition, especially in Western countries, while it is more frequently detected in East Asian countries, especially in Japan. In fact, the incidence is estimated to be about 10 times lower in Europe than in Japan, where epidemiological research found an incidence of 0.35–1.13/100,000 per year [44]. We refer to “Moyamoya disease” (MMD), when the “Moyamoya phenomenon” presents as an isolated angiopathy. On the other hand, Moyamoya angiopathy may be associated with other clinical manifestations, neurological or extra-neurological; these situations are known as “Moyamoya syndrome” (MMS). The age of clinical onset follows a biphasic distribution. The first peak occurs between 5 and 19 years, and the second peak occurs during the fourth decade of life [43]. The clinical presentation of MMD varies with age. Typically, in adults the onset consists of ischemic or hemorrhagic stroke, while in children ischemic events are more common. It is estimated that about 20% of strokes in children with Moyamoya disease are hemorrhagic while the remaining 80% are ischemic [45]. Therefore, the main clinical features are due to stroke, such as hemiparesis, dysesthesia/hypoesthesia or alterations in speech. Less frequently, other kinds of symptoms may be present: alteration in sight, syncope or psychiatric disequilibrium. Other possible clinical manifestations can be ascribed to chronic cerebral hypoxia, such as intellectual disability, which may be found in children (especially in those who have experienced a stroke) and headache, which is typically migraine-like. Moyamoya angiopathy’s pathogenesis is not entirely clear, although genetics appears to play a key role, as witnessed by an often-positive family history. In addition, it is associated with some genetic disorders, as well as some variants of the *RNF213* gene [46]. In fact, MMS can be secondary to some hereditary diseases, such as neurofibromatosis type 1, Noonan syndrome, Alagille syndrome, sickle cell disease, Down syndrome, Turner syndrome and PHACE syndrome. In addition, it is reported in subjects with homozygous variants of SAMHDI, MOPDII and GUCY1A3 [47]. The GUCY1A3 variant is a rare neurological disease characterized by the association between neonatal-onset achalasia and Moyamoya angiopathy, often together with hypertension. These individuals typically present with stroke and coronary artery disease (CAD) [48]. Also, the ACTA2 variant in heterozygosity can lead to a broad picture of vascular pathology, which includes MMD, along with aortic aneurysms, aortic dissection and premature CAD [49].

### 4.3. COL4A1

COL4A1 encodes the α1-chain of type IV collagen, which ubiquitously composes basement membranes. The COL4A1 variant is a rare condition, as less than 100 families with the variant have been described in the literature, with an autosomal dominance inheritance type. The onset, type and severity of clinical manifestations are heterogeneous and vary between different families and within the same family. Patients may be asymptomatic or present with neurological manifestations (porencephaly, lacunar ischemic stroke, intracranial hemorrhage including microbleeds, cerebral aneurysms, childhood-onset seizures, intracerebral calcifications, hemiplegia, migraines, dementia and intellectual disability), eye defects (congenital cataract, retinal arteriolar tortuosity, anterior chamber abnormalities of the Axenfeld–Rieger type) and other clinical features (microcephaly, muscle cramps, serum CK elevation, renal involvement with possible hematuria, Raynaud phenomenon, cardiac arrhythmia, hemolytic anemia) [50]. Since type IV collagen is contained in vessel walls, variants can result in hemorrhage, with the brain being the most common site of occurrence [51]. Intracerebral bleeding may occur at any stage of life, including before birth (later resulting in porencephaly) [52], neonatal age and childhood; in addition, patients are likely to experience recurring episodes [20]. It can occur either spontaneously or following minor trauma and it can range from microbleeds to massive intracranial hemorrhage [53]. According to Whittaker et al., stroke is the most common cerebral manifestation in COL4A1-mutated patients, and in most cases, it is the hemorrhagic type [54]. Sometimes, hemorrhagic strokes and other fetal abnormalities might be found in prenatal ultrasound and then confirmed after birth [55]. Lafranconi et al. in their systematic review reported white matter hyperintensities on MRI scans in patients aged from 7 days to 68 years, while the mean age of hemorrhagic stroke was around 32 years (range 14–49 years old) [56].

### 4.4. COL4A2 (Familial Porencephaly)

The COL4A2 gene encodes the α2-chain of type IV collagen, which is a structural element of the basement membrane. Its pathogenetic variant, with a dominant autosomal transmission, leads to changes in the structural properties of vessels, and is associated to familial porencephaly. This disease is characterized by the presence of brain cysts, together with a cerebral hemorrhage in neonatal or perinatal time. It is often associated with the presence of other brain abnormalities, such as hydrocephalus, silent periventricular leukoencephalopathy, calcifications and aneurysms [18,57]. The clinical features associated with this condition are highly varied. They may include neurological manifestations such as stroke, seizures, migraine and infantile hemiparesis. Additionally, systemic symptoms may also be present, involving different organs (cataract, cardiac abnormalities, nephropathy with urinary retention, muscle alterations) [52]. This is well pictured by the study lead by Hausman-Kedem et al., in which the different phenotype presented by members of the same family, all with COL4A2 deletion, is described. Neurological symptoms varied: some subjects were asymptomatic, others hemiplegic with cerebral palsy. Manifestations at neuroimaging also ranged widely, including hemorrhagic lacunar infarct, prenatal IVH, porencephaly, microbleeds and hyperintensity in T2-weighted sequences [58]. Therefore, COL4A2 variants are associated with fetal and neonatal hemorrhages and spontaneous recurrent intracranial hemorrhages. The variant also leads to increased susceptibility to intracranial hemorrhage following trauma.

### 4.5. Ehlers–Danlos Syndrome

Ehlers–Danlos syndrome type IV, alternatively known as vascular Ehlers–Danlos syndrome, is an autosomal dominant condition due to a variant in the COL3A1 gene. This gene codes for procollagen III and its variant leads to an alteration in the structure of the protein with a reduced elasticity and strength of collagen, which is part of the visceral connective tissue and blood vessel wall. The disease is usually diagnosed in children due to a positive family history; in fact, the clinical onset typically occurs in adult age. Clinically, it is characterized by alterations in facial morphology with typical appearance (e.g., thin lips and nose, lobeless ears), dermal dysplasia (the skin presents as extensible, translucid and thin, and subcutaneous vessels might be visible), increased fragility of organs and vessels, which can result in the rupture of organs (uterus and bowel) and in the development of hematomas and ecchymosis. Nevertheless, phenotypical characteristics may not always be obvious, which can result in a lack of diagnosis. Digestive symptoms and obstetric complications are also common [59]. Vascular alterations are a common manifestation and mainly affect medium- and large-caliber vessels, and they might lead to a tendency to spontaneous bruising since early age. Cerebrovascular manifestations are not rare. North et al. estimated that they might be detected in about 10% of E-D type IV patients [60]. In these patients, alterations of arterial vessels may be found. In addition, Ehlers–Danlos patients present an increased risk of carotid-cavernous fistulae, intracranial aneurysms (especially in the cavernous sinus) [61], subarachnoid hemorrhage and spontaneous dissection or rupture of intracranial arteries. The rupture of aneurysms is possible, and it may occur spontaneously or due to physical activity. It is estimated that about 4% of patients present with intracranial hemorrhages [21]. In Ehlers–Danlos patients type IV, premature death usually occurs due to arterial rupture or dissection [62]. Shur et al. reported a case of a 4-month-old child with Ehlers–Danlos syndrome type IV (she tested positive for a variant of unknown significance), who had subdural hemorrhages, together with retinal hemorrhages and bone fractures. The baby’s history was negative for trauma [9]. Neurovascular manifestations of the disease are also possible in other types of Ehlers–Danlos, even in childhood. Quade et al. report a case of a 13-year-old female with EDS type VI (kyphoscoliotic type), due to the PLOD1 variant, who reported a stroke. At birth, she had a small hemorrhage in the white matter, together with other symptoms, such as generalized hypotonia and skin hematomas. During the following years, the perinatal intracranial hemorrhages progressed with gliotic changes in white matter at MRI scans [25].

### 4.6. Neurofibromatosis Type 1 (Nf1)

Neurofibromatosis type 1 (Nf1) is a neurocutaneous disorder caused by an autosomal dominant variant of the *NF1* gene, with alteration of its encoded protein product neurofibromin [63]. Clinically, it is characterized by the presence of cafè-au-lait skin spots, axillary freckling, cutaneous and plexiform neurofibromas, Lisch nodules (iris hamartomas), bone alterations (including bone dysplasia) and an augmented risk of developing certain neoplasms (such as optic pathway gliomas and pheochromocytomas). Moreover, hypertension is more frequently found in affected individuals, and also in children. In addition, patients with neurofibromatosis type 1 have been observed to present with several types of vascular lesions, including stenosis, aneurysms, ectasia, fistulae and vascular rupture. The pathogenesis of vasculopathy in NF1 is not fully understood, but it is thought to be linked to the role of neurofibromin in blood vessels. In fact, this protein is expressed in both smooth muscle and endothelial cells [64]. A small percentage of Nf1 patients (lower than 5%) are affected by cerebral vasculopathy. It includes a variety of alterations in intracranial arteries, which at first could be asymptomatic. They comprise ectasia, stenoses and aneurysms. Furthermore, a Moyamoya pattern may be found, with the development of a fragile new collateral vascular net and possible bleeding. Clinically, they can present as ischemic stroke and intracranial bleeding [28]. Therefore, Nf1 leads to an augmented risk of stroke compared to the general population. This is dramatically higher when considering the hemorrhagic stroke subtype and children’s stroke. According to a study conducted by Terry et al., the risk of developing hemorrhagic stroke in children (including SAH and ICH) was 7 for every 1000 Nf1 patients, while in the general population it was only 0.5 every 1000 children (OR 6.3, with an OR of 8.1 for ICH) [27]. Rosser at al. retrospectively analyzed Nf1 patients and found eight cases of cerebral vasculopathy. Most of them only had incidental findings, but the radiological features were variate, including stenosis, occlusion, ectasia, infarcts, aneurysm and Moyamoya pattern. Among them, an ICA asymptomatic aneurysm was found in a 1-year-old child and an asymptomatic Moyamoya pattern was detected in a 2-year-old patient. In addition, a patient aged 2 years old experienced acute hemiparesis due to infarcts and Moyamoya disease [28]. The study led by Rea et al., found at least a 6% prevalence of cerebral arteriopathy in children with NF1. They reported different kinds of cerebral vascular complications in children, including intracranial hemorrhage [29].

### 4.7. Sickle Cell Disease (SCD)

SCD is an inherited hemoglobinopathy caused by a usually homozygous variant in the β-globin gene (HbSS), with autosomal inheritance, which causes an abnormality of the chain structure with a reduced deformability of erythrocytes. This results in an increased viscosity of the blood, and consequently in episodes of blood vessel occlusion. It is one of the most widespread genetic diseases across the globe and the highest prevalence is found in sub-Saharan Africa [65]. The most severe complications of sickle cell disease (SCD) affect the central nervous system and they include ischemic and hemorrhagic stroke, cerebral sinus thrombosis, epilepsy, reversible encephalopathy syndrome and silent cerebral infarct (SCI) [66]. These neurologic complications usually manifest as migraine and recurrent headaches (SCD must be suspected if these clinical manifestations are detected in a child). In fact, the rates of stroke are much higher in SCD children and adults than in the general population, especially among children aged from 2 to 5 years old, in which it is estimated from 790 per 100,000 persons-years [67]. Focusing on intracranial bleeding, even if it is not a frequent complication in SCD, it is about 200 times more frequent in SCD patients than in the general population: it is estimated to be more common in patients from 20 to 29 years of age, but the rate in the above-mentioned age range (2–5 years old) is 150 per 100,000 persons-years. The mortality rate is high in both adults and in children (from 26% to 65% in the first 2 weeks after the hemorrhagic stroke); in fact, it represents the main mortality cause in SCD patients [67]. In the literature, cases of both massive intracranial hemorrhages and delayed intracranial hemorrhages following cerebral infarction have been described [68,69]. Patients with SCD tend to develop intracranial aneurysms more frequently than the general population, probably due to cerebral vasculopathy; these vascular malformations tend to be smaller in size but with a higher tendency to bleed. In fact, the most frequent cause of intracranial hemorrhage in SCD patients is the bleeding of an intracranial aneurysm [70]. In addition, SCD is one of the congenital diseases that can lead to Moyamoya disease, which can eventually lead to bleeding of the fragile basal collaterals (see Section 4.2), resulting in intracranial hemorrhage [71]. Furthermore, intracranial bleeding may be secondary to hypertension or the dysfunction of cerebral vessel autoregulation. Some risk factors for hemorrhagic stroke have been identified, such as hypertension, renal disease, coagulopathy, supraventricular arrhythmia and RBCs transfusion (during the past 2 weeks). Additionally, some drugs (e.g., corticosteroids, NSAIDs) and some features of SCD (such as a low steady state of hemoglobin) seem to increase the risk of intracranial bleeding in these patients [66]. Al-Jarallah et al. analyzed 68 children who experienced nontraumatic intraparenchymal hemorrhage. Among them, three had SCD (aged 6, 4 and 2.5 years old) and in at least one case the ICH followed an ischemic infarction [30]. In conclusion, primarily hemorrhagic stroke is not a common complication in SCD patients, but it may also occur in children younger than 5 years old and it is burdened with a high risk of mortality [72].

### 4.8. Cerebral Cavernous Malformations (CCM)

Cerebral cavernous malformations (CCMs) are lesions involving central nervous system vessels, in which the capillary lumen is excessively enlarged and the wall is thinned [73]. Radiologically, CCM lesions appear in MRI T2-weighted sequences as the typical “popcorn”-like image. It is not a rare disorder, as it is present in about 1 to 5 in every 1000 people [74]. Generally, the occurrence of CCM is sporadic; however, hereditary forms, due to the germline variant, are also possible, often involving multiple vascular formations. Familiar CCM is mainly caused by a heterozygous loss-of-function variant in one of the following genes: *KRIT1* (CCM1), *MGC4607* (CCM2) and *PDCD10* (CCM3) [75]. Frequently, the diagnosis is incidental, and the clinic is asymptomatic. Nevertheless, the clinical picture may include seizures, hemorrhagic strokes, focal neurologic signs (unrelated to bleeding events) and headaches. Intracranial hemorrhage is estimated to be present in approximately from 25 to 40% of cases [76]. The average age of clinical onset is around 36 years of age, but the range includes neonates, as well as older patients [77]. Spiegler et al. reported a case of an 18-month-old boy who tested positive for a heterozygous CCM1 variant and had multiple cavernomas, some of which were bleeding [23]. In a paper by Sürücü et al., a case is reported of a 3-year-old girl, CCM1-mutated, with growing and bleeding brainstem cavernomas experiencing intracranial hemorrhage and neurological deterioration [24].

### 4.9. Hereditary Hemorrhagic Telangiectasia/Osler–Weber–Rendu Syndrome

Hereditary hemorrhagic telangiectasia (HHT), which is also known as Osler–Weber–Rendu syndrome, is a genetic disease, characterized by the presence of organ arteriovenous malformations (AVMs). AVMs are a direct connection between the artery and the vein, lacking the interposed capillaries, and can be present in multiple organs, including mucous membranes, internal organs and skin (face, fingers). The most relevant ones in size are usually found in internal organs such as brain, liver or lungs [78]. Clinically, the first symptom in HHT patients is usually spontaneous epistaxis, which begins at around 12 years of age. Patients may also present with mucocutaneous telangiectasias on lips, tongue, gastrointestinal system mucosa or on the skin (e.g., face, hands). In addition, due to the fragility of the vascular wall, AVMs can bleed, leading to mild to severe anemia and different clinical manifestations, depending on which organ is affected, including acute and potentially catastrophic complications. In fact, AVMs complications represent the main causes of sequelae in HHT patients [79]. Cerebral AVMs, which are present in about 10% of HHT cases, are often already detectable at birth. Frequently, they are multiple, and typically manifest with hemorrhage. According to a meta-analysis conducted by Brinjikjiet al. in 2017, more than half of AVMs are symptomatic and cerebral hemorrhage is the first manifestation in about 20% of patients with HHT [80]. According to Saleh et al., the age of diagnosis of AVMs is on average 8.91 years, but the range varies between 0.6 and 18 years. They also report ICH due to the rupture of cerebral AVM in children of different ages, some of which were newborns (two 9-month-old babies and one 1.7-year-old baby), some of them with epistaxis in previous years [32]. Furthermore, the mean age of intracranial hemorrhage in patients with HHT is 25 years, but bleeding due to the rupture of cerebral AVMs is also described in patients of a younger age. Morgan et al. report several cases of HHT patients: one with ICH in neonatal age, along with eight cases of ICH in children. Some of them resulted in a fatal outcome and were confirmed by autopsy [31]. Therefore, HHT should be promptly suspected in patients presenting with frequent nosebleeds, multiple telangiectasias or if visceral AVM are detected. Additionally, a positive family history of HHT or the clinical features are suggestive of HHT [1]. Monogenic variants that lead to the development of HHT are transmitted by autosomal dominant inheritance, and the major loci involved are ACVRL1 (55%), ENG (44%) or SMAD4 (1%). Patients with the latter variant present with a combined syndrome (HHT associated with juvenile polyposis) [81].

### 4.10. Marfan Syndrome

Marfan syndrome is a genetic disease, with an autosomal dominant transmission, due in most cases to a variant of the gene coding for fibrin-1 (*FBN1*), which composes microfibrils, proteins found in the extracellular matrix. Mutated microfibrils result in faulty links between elastic fibers and the smooth muscle cells of blood vessels. Therefore, clinical manifestations arise from impaired connective tissue and involve multiple systems, with typical skeletal symptoms (long limbs, pectus excavatum or carenatum, scoliosis, laxity of ligaments with hyperextensible joints), alterations in the cardiovascular system (affecting both heart and vessels) and ocular manifestations (ectopia lentis, cataract, strabismus). Regarding cardiovascular manifestations, the most common ones are aortic aneurysms and dissection. Vascular changes are due to the alteration of fibrin, which is found in the wall of arteries; therefore, in patients with the *FBN1* variant, vessels become less resistant. Neurovascular manifestations can be observed, but they are less common [82]. There are several explanations for neurovascular changes; they are often due to an extension of aortic dissection [20]. Additionally, it is commonly believed that patients with Marfan syndrome are at a higher risk for intracranial aneurysms, even in childhood [83]. Wityk et al. wrote a case series about the neurovascular complications of Marfan syndrome. Among these, they reported a female child, aged less than 1 year old, with Marfan syndrome who presented with subdural hematoma. She also had aortic and mitral valve prolapse and was on anticoagulation treatment. Nonetheless, according to Wityk et al., patients with Marfan syndrome who have neurovascular disorders are typically older than those without these complications [26].

### 4.11. Role of Micro RNAS in Hemorrhagic Stroke

MicroRNAs (miRNAs) are small non-coding RNA molecules naturally present in our body and stable both inside and outside cells. In recent years, particular attention has been paid to the study of microRNAs as potential biological markers useful for the diagnosis, treatment and prognosis of cerebrovascular diseases. It has been observed that some miRNAs are closely associated with hemorrhagic stroke, caused by various conditions such as intracerebral hemorrhage, intracranial aneurysms and subarachnoid hemorrhage. These miRNAs play a crucial role in regulating the molecular processes that occur after hemorrhagic stroke. The diagnosis and treatment of hemorrhagic stroke can benefit from the information provided by the stroke-miRNA system. During the expansion of intraparenchymal hemorrhage, the identification of specific miRNAs may be useful in assessing the risk of further complications. In patients with intraparenchymal hemorrhage, hemorrhage expansion is the main cause of mortality and functional impairments [84]. The low expression of certain miRNAs, such as miR-126, has been associated with an increase in perihematomal edema, which can lead to severe consequences [85]. Some miRNAs, such as miR-130a, can serve as non-invasive markers for cerebral edema and can be used to predict disease progression [86]. These miRNAs influence the permeability of the blood–brain barrier, thus contributing to the formation of cerebral edema. Moreover, various miRNAs, such as miR-124, which decreases in ischemic stroke, play a role in the pathophysiology of intracerebral hemorrhage, influencing both the inflammatory and anti-inflammatory responses in the brain. In the case of subarachnoid hemorrhage due to a ruptured aneurysm, AVM or cranial trauma, miR-502-5p, miR-1297 and miR-4320 can be used as diagnostic and prognostic indicators of the severity of the condition [87]. Furthermore, for post-subarachnoid hemorrhage arterial vasospasm, miR-3177-3p has been identified as a potential biomarker [88]. The study of miRNAs can serve as a valuable adjunctive tool for diagnosing and prognosticating cerebrovascular diseases. Acquiring knowledge about miRNAs can assist clinicians in gaining a deeper understanding of the ongoing pathological process and in devising more tailored treatment strategies.

### 4.12. Multidisciplinary Approach in the Diagnosis of Non-Accidental Trauma

Infant cranial trauma is a sensitive topic in both the clinical and forensic fields, requiring careful assessment to determine its cause and ensure appropriate treatment. It is crucial to recognize that not all pediatric cranial traumas result from accidental incidents; in some cases, there may be non-accidental causes that require thorough management and specific intervention. Therefore, an algorithmic approach could prove extremely useful in diagnosing cases of NAT. During the initial phase, a detailed medical history should be collected, focusing on any history of incidents, signs of abuse or suspicious injuries. It is important to involve parents or caregivers in the process to obtain accurate and complete information, as suspicion often arises from their unreliable or contradictory accounts [89]. Documenting the caregiver’s abuse of alcohol or drugs, psychiatric illnesses or a history of criminal activity or domestic violence may be revealing [90]. The clinical examination should be comprehensive and targeted, with particular attention to identifying signs of cranial trauma, including bruising, contusions, burns, fractures or signs of internal bleeding. One of the most feared manifestations is AHT or shaken baby syndrome, which primarily affects children under 2 years old and is often characterized by fatal brain injuries. Children may be asymptomatic or may present with symptoms such as nausea, vomiting, poor appetite, irritability, lethargy, bleeding, headache or seizures. Although this condition is characterized by a classical triad (subdural hemorrhage, retinal hemorrhage and encephalopathy), it may be associated with fractures, skin lesions (burns, bruises, lacerations) or even more figurative signs (e.g., marks of fingers or means of abuse) [91]. Suspicious skin injuries are those that a child would hardly accidentally cause (facial, neck, buttock injuries) [92]. Sometimes the trauma is so severe and widespread that it may also result in abdominal injuries, epidural or subarachnoid hemorrhages and cranial fractures (multiple, depressed, diastatic, stellate or ping pong) [90]. The latter are predominantly found in children under 2 years old, where cranial ossification is insufficient. Parenchymal injuries may be the most complex to evaluate, as they may not always present typical signs of AHT such as cerebral contusions, axonal injuries and intracranial hemorrhages. When the trauma affects the brainstem, there may be direct trauma to the vital centers resulting in diffuse hypoxic-ischemic injuries [93]. If the newborn survives and there is a long history of abuse, acute NAT signs may overlap with chronic ones, which may result in multicystic encephalopathy [94]. Radiological evaluation is a crucial step in identifying cranial injuries. Skull radiography is often the first step, but it may be followed by computed tomography (CT) or magnetic resonance imaging (MRI) depending on the severity of the condition or suspected internal injuries. Radiological images should be carefully evaluated to detect signs of fractures, hematomas, cerebral edema or other anomalies suggestive of non-accidental trauma and to assess other body areas (chest, abdomen and limbs) in the presence of suspicious injuries [10]. In cases of multiple posterior–anterior or metaphyseal rib fractures or other fractures that are difficult to explain, a multidisciplinary approach should be adopted. Laboratory tests such as hematocrit, coagulation studies, electrolytes and liver function should not be overlooked. If a genetic condition is clinically suspected, further investigation should be pursued. The evaluation of retinal hemorrhages in vivo requires the intervention of an experienced ophthalmologist who can assess the spread of hemorrhage within the retinal layers [95,96]. Once suspicious injuries are identified, it is important to involve a multidisciplinary team comprising pediatricians, neurologists, radiologists, ophthalmologists, social workers and forensic experts [97]. This team will collaborate to assess the clinical, social and family context of the child, exclude other causes of injuries, and determine whether there are signs of abuse or neglect. The social worker may play a key role in assessing family dynamics and identifying any concerns regarding the child’s safety. Throughout the investigation, it is essential to thoroughly document all collected information, including radiological images, clinical observations and multidisciplinary discussions. A detailed report should be drafted and shared with all team members involved in the case, as well as with relevant authorities if necessary. Once non-accidental causes of cranial trauma are identified, it is essential to plan appropriate follow-up for the child and their family. This may include the involvement of support services, psychological counseling and interventions to ensure the long-term safety and well-being of the child. Additionally, legal proceedings may need to be initiated or assistance provided in accessing child protection services, if appropriate.

## 5. Limitations

The current study exhibits certain limitations. First, the number of papers about cases of children with intracranial hemorrhage who underwent genetic investigation was a restricted quantity. Moreover, a primary limitation arose from numerous studies failing to specify the stroke subtype (hemorrhagic or ischemic) and/or the patient’s age.

Additionally, while we extensively searched publicly available databases (PubMed and Google Scholar), we acknowledge the possibility that there might be additional relevant articles that were not encompassed within the selected databases. Moreover, the studies incorporated in the current study exhibit a notable heterogeneity in the study design.

Furthermore, another persistent limitation of the study is our exclusive inclusion of English-language publications.

## 6. Conclusions

**Figure 2:**
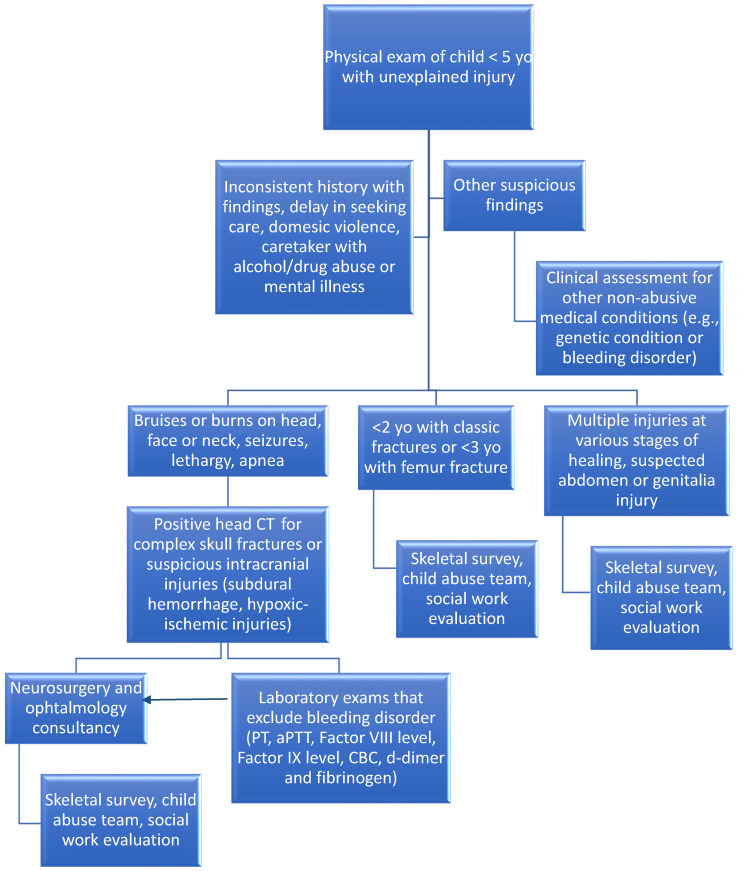
The proposed algorithm that should guide physicians in cases of suspicious AHT.

This article could serve as a potential roadmap for researching genetic conditions associated with unspecified pediatric hemorrhagic stroke. In cases where there is doubt and suspected NAHI, the presence of genetic disorders cannot automatically be used to support spontaneous intracranial hemorrhages. Therefore, in suspicious cases, it would be advisable to proceed with a diagnostic protocol [97,98] and a multidisciplinary assessment (Figure 2). Multidisciplinary assessment is crucial to substantiate NAHI in a child with a genetic predisposition that inherently increases the risk of bleeding. It is important to investigate all the injuries without neglecting the potential hypothesis of NAHI in patients with genetic diseases. This applies to cases where death occurs shortly after trauma as well as cases where there is a significant post-mortem interval following hemorrhagic stroke, lasting many years.

## Figures and Tables

**Table 1:** Clinical features in abusive trauma.

CLINICAL FEATURES SUSPICIOUS OF ABUSIVE TRAUMA
♦ Classic triad: subdural hematoma (SDH), cerebral edema or encephalopathy, retinal hemorrhage (RH); ♦ Hypoxic-ischemic injuries and spine-related injuries without traumatic history; ♦ Multiple fractures (especially when in different stages of healing) or complex skull fracture (multiple, stellate, crossing suture lines, depressed, bilateral, ping pong fracture); ♦ Location of fractures: posterior rib, long bone, metaphyseal corner fractures, sternal fracture (especially in <1 years old), femur fracture in children <3 y old; ♦ Abrasions and/or bruising in atypical anatomical sites (face, ear, neck, torso, buttocks, genitals, back), particularly suspicious if in various stages of healing or when the child is in preambulatory stage; ♦ Patterned bruises (loop, belt, hand) or bite marks; ♦ Associated burns (immersion or branding burns); ♦ Bulging fontanel; ♦ Indicators of neglect (in clothing, general hygiene); ♦ Other clinical features: seizures, irritability, lethargy/mental status alteration, respiratory distress, vomiting, reduced oral intake, developmental delay of unknown origin.

**Table 2:** Genetic diseases in stroke.

Title	Authors	Year	Genetic Disorders	Only Pediatric Population
Monogenetic Stroke Syndromes in Children and Young Adults	Doig et al. [15]	2020	- *COL4A1*, *COL4A2* - HCHWA-D	NO (children and young adults)
Management of Stroke in Neonates and Children: A Scientific Statement From the American Heart Association/American Stroke Association	Ferriero et al. [1]	2019	- *CCM 1, 2, 3* - *COL4A1* - Hemophilia A, Hemophilia B, von Willebrand disease - HHT - *RASA-1*	YES
Hemorrhagic Cerebrovascular Pathology in the Pediatric Population	Guerrero et al. [6]	2020	AVM: - HHT - RASA-1 - Wyburn-Mason syndrome Aneurysms: - E-D syndrome - FMD - HHT - Klippel–Trenaunay–Weber syndrome - Marfan syndrome - PKD	YES
Monogenic Causes of Cerebrovascular Disease in Childhood: A Case Series	Ostrem et al. [16]	2023	- *ACTA2* - *ACVRL1* - *COL4A1* - *ENG* - *FOXC1* - *KRIT1* - *PDCD10* - *RNASEH2B* - *SAMHD1* - Sturge–Weber syndrome	YES
Genetic Risk Factors for Ischemic and Hemorrhagic Stroke	Chauhan et al. [17]	2016	- CAA - CADASIL - CARASIL - *COL4A1*	NO
Monogenic Causes of Strokes Genetic	Chojdak- Łukasiewicz et al. [18]	2021	- *CCM* - *COL4A1* - *COL4A2* - E-D syndrome - Fabry disease - HCHWA-D - Marfan syndrome - MELAS - PXE - SCD	NO
Intracerebral Hemorrhage Genetics	Ekkert et al. [19]	2022	- CAA - CADASIL - *COL4A1* - *COL4A2*	NO
Single-gene stroke disorders	Majersik et al. [20]	2006	- CADASIL - E-D syndrome - Fabry disease - Marfan syndrome - Moyamoya - SCD - Sneddon syndrome - PKD	NO
The genetic basis of strokes in pediatric populations and insight into new therapeutic options	Jankovic et al. [21]	2022	- *ACTA-2* - CADASIL - CARASAL - *COL4A1* - DADA-2 - E-D syndrome - Fabry disease - Marfan syndrome - MELAS - Menke’s disease - Moyamoya - NF1, NF2 - PXE - Sturge–Weber syndrome - VHL	YES
Genetic and Environmental Associations With PediatricCerebral Arteriopathy	McCrea et al. [22]	2019	- *ACTA2* - *COL4A1* - *COL4A2* - *DADA2* - Moyamoya - *MYH11* - NF1	YES

**Abbreviations**: ACVRL1: activin A receptor like type 1, ACTA2: actin α 2, AVM: Arteriovenous malformations, CAA: cerebral amyloid angiopathy, CCM: cerebral cavernous malformations, CADASIL: Cerebral Autosomal Dominant Arteriopathy with Subcortical Infarcts and Leukoencephalopathy, CARASAL: Cathepsin A-related arteriopathy with strokes and leukoencephalopathy, CARASIL: cerebral autosomal recessive arteriopathy with subcortical infarcts and leukoencephalopathy, COL4A1: collagen type IV α 1 chain, COL4A2: collagen type IV α 2 chain, DADA-2: Deficiency of Adenosine Deaminase 2, ENG: Endoglin, E-D: Ehlers–Danlos, FMD: Fibromuscular dysplasia, HCHWA-D: hereditary cerebral hemorrhage with amyloidosis—Dutch type, HHT: hereditary hemorrhagic telangiectasia, KRIT1: trapped gene 1, MELAS: Mitochondrial encephalopathy, lactic acidosis and stroke-like episodes, MYH11: myosin heavy chain 11, Nf1: Neurofibromatosis type 1, Nf2: Neurofibromatosis type 2, PDCD10: programmed cell death 10, PKD: polycystic kidney disease, PXE: Pseudoxanthoma Elasticum, RASA-1: Ras p21 protein activator 1, RNASEH2B: Ribonuclease H2 subunit B, SAMHD1: sterile α motif and hemidesmosome domain containing protein 1, SCD: sickle cell disease, VHL: Von Hippel-Lindau disease.

## Data Availability

The original contributions presented in the study are included in the article and Supplementary Materials, further inquiries can be directed to the corresponding author.

## Supplementary Materials

The following supporting information can be downloaded at: https://www.mdpi.com/article/10.3390/genes15050618/s1, Table S1: Risk of bias.
